# Influence on [^18^F]FDG uptake by cancer cells after anti-PD-1 therapy in an enforced-immune activated mouse tumor

**DOI:** 10.1186/s13550-020-0608-4

**Published:** 2020-03-19

**Authors:** Mayu Tomita, Motofumi Suzuki, Yusuke Kono, Kohei Nakajima, Takuma Matsuda, Yuji Kuge, Mikako Ogawa

**Affiliations:** 1 Laboratory of Bioanalysis and Molecular Imaging, Graduate School of Pharmaceutical Sciences, Hokkaido University, Sapporo, Hokkaido 060-0812 Japan; 2 Central Institute of Isotope Science, Hokkaido University, Sapporo, Hokkaido 060-0815 Japan

**Keywords:** PD-1, Immune checkpoint inhibitor, [^18^F]FDG, cGAMP

## Abstract

**Background:**

Anti-programmed cell death 1 (PD-1) antibody is an immune checkpoint inhibitor, and anti-PD-1 therapy improves the anti-tumor functions of T cells and affects tumor microenvironment. We previously reported that anti-PD-1 treatment affected tumor glycolysis by using 2-deoxy-2-[^18^F]fluoro-*D*-glucose ([^18^F]FDG) positron emission tomography (PET). That study showed that anti-PD-1 therapy in a mouse B16F10 melanoma model increased glucose metabolism in cancer cells at the point where anti-PD-1 therapy did not cause a significant inhibition of tumor growth. However, the B16F10 melanoma model is poorly immunogenic, so it is not clear how anti-PD-1 treatment affects glucose metabolism in highly immunogenic cancer models. In this study, we used a cyclic dinucleotide GMP-AMP (cGAMP)-injected B16F10 melanoma model to investigate the effect of anti-PD-1 therapy on [^18^F]FDG uptake in a highly immune activated tumor in mice.

**Results:**

To compare the cGAMP-injected B16F10 model with the B16F10 model, experiments were performed as described in our previous manuscript. [^18^F]FDG-PET was measured before treatment and 7 days after the start of treatment. In this study, [^18^F]FDG uptake in tumors in the cGAMP/anti-PD-1 combination group was lower than that in the anti-PD-1 treatment group tumors on day 7, as shown by PET and ex vivo validation. Flow-cytometry was performed to assess immune cell populations and glucose metabolism. Anti-PD-1 and/or cGAMP treatment increased the infiltration level of immune cells into tumors. The cGAMP/anti-PD-1 combination group had significantly lower levels of GLUT1^high^ cells/hexokinase II^high^ cells in CD45^−^ cancer cells compared with tumors in the anti-PD-1 treated group. These results suggested that if immune responses in tumors are higher than a certain level, glucose uptake in cancer cells is reduced depending on that level. Such a change of glucose uptake might be caused by the difference in infiltration or activation level of immune cells between the anti-PD-1 treated group and the cGAMP/anti-PD-1 combination group.

**Conclusions:**

[^18^F]FDG uptake in cancer cells after anti-PD-1 treatment might be affected by the tumor immune microenvironment including immune cell infiltration, composition, and activation status.

## Background

Programmed cell death 1 (PD-1) inhibitors (e.g., nivolumab) are a group of immune checkpoint inhibitors that activate antitumor immune responses. In PD-1 treatment-responsive cancers, anti-PD-1 treatment improves T cell functions and affects the tumor microenvironment (TME) including cancer cells, immune cells, and cancer-associated fibroblasts. In such cancers, immune cells have prolonged, strong anti-tumor effects with less toxicity than conventional chemotherapy [1–3].

Because the mechanisms of anti-PD-1 therapy are different from conventional chemotherapies, anti-PD-1 therapy is characterized by unique points. First, the immunotherapeutic effects of anti-PD-1 treatment occur late, but once they appear, immune responses are maintained long-term [3, 4]. Second, PD-1 inhibitors are not effective in all patients because the therapeutic effects depend on individual-patient immunity and mutation status of cancer genes [5, 6].

These unique points of anti-PD-1 therapy correlate with metabolism in the TME. Recent studies have discussed the correlation between cancer or immune cells and anti-tumor immunity [7, 8]. Chang et al. proposed that glucose consumption by tumors metabolically restricts T cells, thereby allowing tumor progression [9]. Also, Cascone et al. identified tumor glycolysis as a pathway associated with immune resistance in melanoma [10].

It is important to clarify the effect of anti-PD-1 treatment on 2-deoxy-2-[^18^F]fluoro-*D*-glucose ([^18^F]FDG) uptake to evaluate the value of [^18^F]FDG for monitoring the effects of anti-PD-1 therapies. We previously reported that anti-PD-1 treatment affected tumor glycolysis by using [^18^F]FDG positron emission tomography (PET) [11]. That study showed that anti-PD-1 therapy in a mouse B16F10 melanoma model increased glucose metabolism in cancer cells at the point where anti-PD-1 therapy did not cause significant inhibition of tumor growth. B16F10 melanoma model was generally used for cancer immunology research because nivolumab was therapeutic agent for melanoma. However, the B16F10 melanoma model is poorly immunogenic compared with CT26 or RENCA models [12, 13], so it is not clear how anti-PD-1 treatment affects glucose metabolism in highly immunogenic cancer models.

Here, we focused on cyclic dinucleotide GMP-AMP (cGAMP), which can cause the enforced activation of stimulator of IFN genes (STING) and enhance antitumor CD8 T responses that control the growth of injected tumors [14, 15]. In this study, we used a cGAMP-injected B16F10 melanoma model to investigate the effect of anti-PD-1 therapy on [^18^F]FDG uptake in highly immune activated tumors. The aim of this study was revealing the effect of anti-PD-1 treatment on tumor glycolysis in a highly immune-activated tumor, by comparing non immunogenic model with enforced immune activation model.

## Materials and methods

### Cell line

The B16F10 melanoma cell line was purchased from ATCC (Manassas, VA) in 2016. Cells were cultured in Dulbecco’s modified Eagle medium supplemented with 10% fetal bovine serum (FBS, Gibco Life Technologies, Grand Island, NY), 1% penicillin-streptomycin, and 2 mmol/L l-glutamine in a humidified incubator at 37°C in an atmosphere of 95% air and 5% carbon dioxide.

### Mouse models

**Fig. 1:**
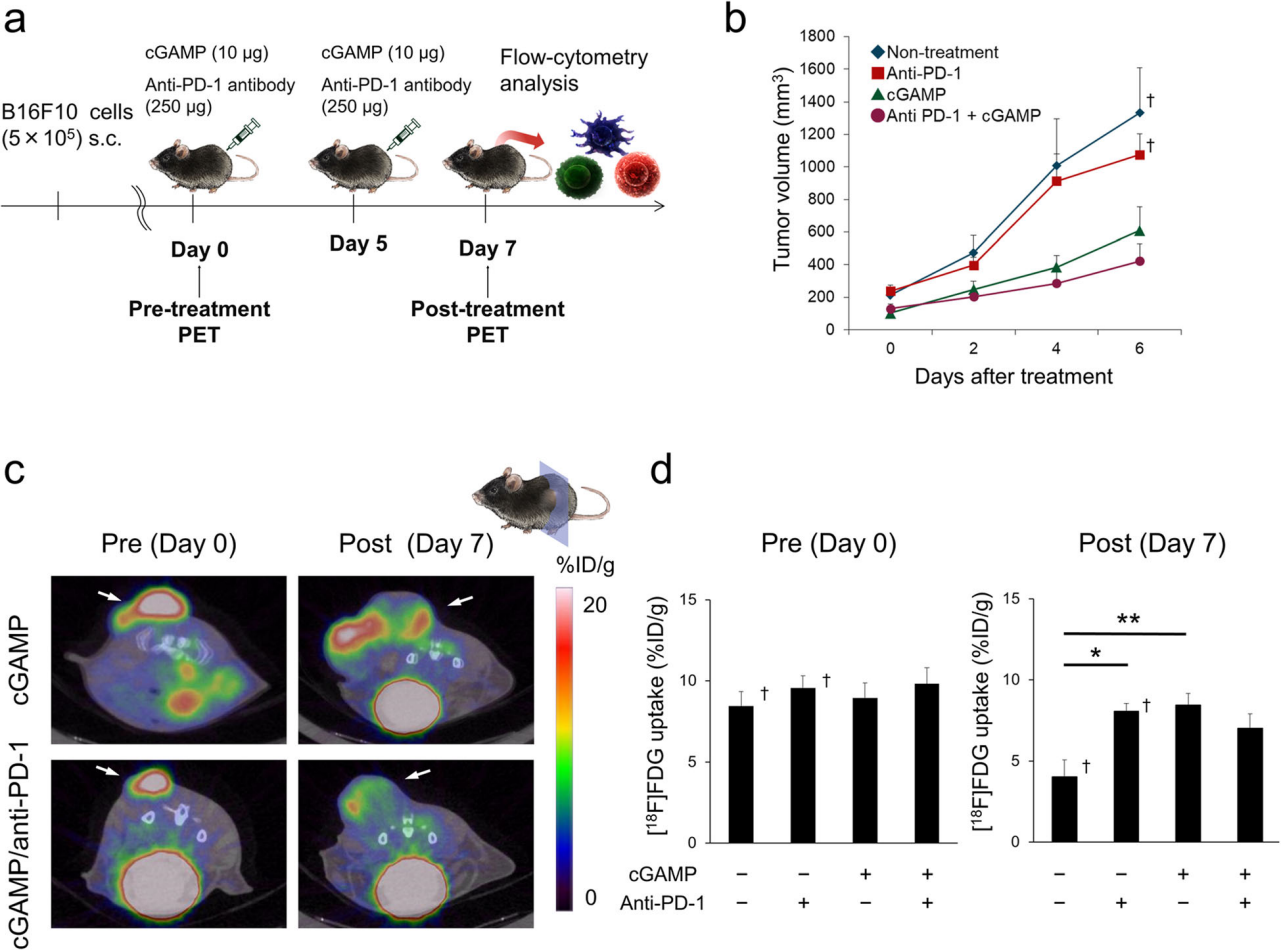
[^18^F]FDG uptake after anti-PD-1 treatment in cGAMP injected mice***.*a** Illustration of in vivo and ex vivo study timelines. **b** Tumor growth curves after anti-PD-1 or cGAMP treatment (*n* = 14–27). Data of the non-treatment group and anti-PD-1 treated group were quoted from [11]. **c** Coronal images from [^18^F]FDG-PET/CT scans from cGAMP alone treated (top) or cGAMP/anti-PD-1 combination treated (bottom) mice on days 0 and 7. **d** [^18^F]FDG uptake was calculated by PET-CT images in tumors on days 0 and 7 (*n* = 6–7). Data of the non-treatment group and anti-PD-1-treated group were quoted from [11]. Data represent the mean ± SEM; *p < 0.05; **p < 0.01; † Adapted from [11]

Animal care, experiments, and euthanasia were performed in accordance with protocols approved by the Hokkaido University Animal Research Committee. Male C57BL/6JJmsSlc mice (7–10 weeks old) were purchased from Sankyo Labo Service Corporation, Inc. (Tokyo, Japan). Mice were inoculated subcutaneously with 5 × 10^5^ B16F10 cells in 100 μL of phosphate-buffered saline (PBS). Anti-PD-1 treatment and cGAMP treatment were started when the presence of tumors (10–15 days after tumor cell induction) was confirmed as day 0 (Fig. 1a). Anti-mouse PD-1 antibody (clone RMP1-14) was purchased from BioXcell (West Lebanon, NH). Anti-PD-1 antibody (250 μg) was administrated twice intra-peritoneally (i.p.) 5 days apart according to the previous methods [11, 16, 17]. On days 0 and 5, tumors were injected with 10 μg of 3′3′-cGAMP (Invivogen, San Diego, CA) complexed with 3 μL of Lipofectamine 2000 (Invivogen) was injected into the tumor [15]. Tumor measurements were made two to three times weekly using calipers, and the volume was expressed in mm^3^ [0.5 × L × W^2^] (L, long diameter; W, short diameter of the tumor). [^18^F]FDG-PET/computed tomography (CT) imaging was performed just prior to initiating therapy and at 7 days after the initiation of anti-PD-1 treatment. The end-point was when the tumor size reached 10% of the body weight.

In this study, tumor volumes were measured in mice and used for PET study or flow-cytometry analysis. To compare the cGAMP-injected B16F10 model with the B16F10 model, data of the non-treatment group and anti-PD-1 treated group were quoted from the previous report [11].

### PET imaging

Thirteen mice were used for PET, ex vivo gamma counting, autoradiography, and staining (cGAMP alone group *n* = 6, cGAMP/anti-PD-1 combination group *n* = 7). The PET study was performed as described in our previous manuscript [11]. Briefly, [^18^F]FDG (3.5 MBq) in 100 μL of saline was administrated to C57/BL6 mice via a lateral tail vein and [^18^F]FDG-PET/CT images were acquired on an Inveon small-animal multimodality PET/CT system (Siemens Medical Solutions, Knoxville, TN). CT scanning was performed from 20 min after the tracer injection, and PET scanning was performed for 10 min beginning at 40 min after the tracer injection.

Acquired PET-CT images were reconstructed using the filtered back projection (FBP) algorithm. PET-CT images and three-dimensional regions of interest (volume of interest; VOI) of tumors were computed using Inveon Research Workplace software (Siemens Medical Solutions). All radioactivity concentration values were normalized according to the percentage-injected dose per gram of tissue (%ID/g), and the mean %ID/g value obtained in VOI was considered for quantitative analysis.

Defining the metabolic tumor volume (MTV) and total lesion glycolysis (TLG) was performed as previously described [18–20]. To exclude the necrotic regions of the tumor, MTV was defined as the VOI where the [^18^F]FDG metabolism was at least 30% of the maximum activity, and mean [^18^F]FDG uptake in MTV was measured as the mean_30%_. TLG was defined as the product of MTV and mean_30%_. Previous data quoted from [11] was reanalyzed to measure the mean_30%_ and TLG.

After PET imaging on day 7, the mice were sacrificed and their organs were dissected. Tissues (tumors, spleens, and blood) were weighed, and radioactivity was measured using a gamma counter (2480 Wizard 2 gamma counter, PerkinElmer, Waltham, MA). Data were calculated as %ID/g.

### Histopathology and autoradiography

Histopathology and autoradiography were performed as described in our previous manuscript [11]. After PET imaging on day 7, serial 5-μm tumor slices were used for autoradiography and hematoxylin-eosin (HE) staining. Autoradiograms were obtained using a phosphor imaging system (FLA-7000, Fujifilm, Tokyo, Japan).

### Flow-cytometry analysis

Thirteen mice were used for flow-cytometry (cGAMP alone group *n* = 6, cGAMP/anti-PD-1 combination group *n* = 7). Tumors were harvested and processed using Collagenase I and DNase I (Wako, Osaka, Japan). The resulting cell suspensions were clarified using 40-μm filters to prepare single cell suspensions, and single cells were suspended in PBS supplemented with 2% FBS. Splenocytes were hemolyzed and incubated with anti-CD16/32 2.4G2 antibody (BD Biosciences, San Jose, CA) to reduce FcγR binding. Cell-surface antigens were stained with antibodies specific for CD8 (BioLegend, San Diego, CA, clone 53-6.7), CD4 (BioLegend, clone GK1.5), and CD45 (BioLegend, clone 30-F11).

For intracellular staining, cells were fixed and permeabilized using a Foxp3/Transcription Factor Staining Buffer Set (eBioscience, San Diego, CA) after cell surface staining and then stained with labeled antibodies against the intracellular molecules Foxp3 (eBioscience, clone FJK-16s), glucose transporter 1 (GLUT1, Abcam, Cambridge, UK, clone EPR3915), and hexokinase II (HX2, Abcam, clone EPR20839).

Samples were analyzed on a FACS Calibur HG flow cytometer (BD Biosciences). Data analysis was performed with CellQuest™ software (Becton Dickinson, Lincoln Park, NJ).

### Immunohistochemistry

Five-micrometer tumor slices for immunohistochemical staining were treated with 3% H_2_O_2_ to quench endogenous peroxidase, and then further blocked with a 10% goat serum (Wako). Slices were stained with rabbit anti-mouse CD3 antibody (Abcam, clone SP7) at a 1:100 dilution. CD3^+^ T cells were detected using Peroxidase-conjugated goat anti rabbit IgG (1:500; Jackson ImmunoResearch, West Grove, PA). DAB tablet (Wako) was used for staining color development, and the counterstaining was performed with hematoxylin solution.

### Statistical analyses

Statistical analyses were performed using JMP pro 14 software (SAS Institute Inc., Cary, NC). Results are expressed as the mean ± SEM. Differences were evaluated by one-way ANOVA. Statistical significance was determined by the Tukey-Kramer test, and *P* values less than 0.05 were considered statistically significant.

## Results

### In vivo [^18^F]FDG-PET imaging of anti-PD-1 treated tumors in cGAMP-injected B16F10 mice

cGAMP only or cGAMP/anti-PD-1 combination treatment was highly effective at inhibiting B16F10 tumors compared with anti-PD-1 treatment alone or non-treatment (Fig. 1b). PET-CT images and [^18^F]FDG uptake values on days 0 and 7 are shown in Fig. 1c, d. [^18^F]FDG scans on day 7 indicated the decreased accumulation by cGAMP/anti-PD-1 combination treatment compared with anti-PD-1 treatment alone, although the difference was not statistically significant (Fig. 1d). The mean_30%_ and TLG on day 7 had a similar tendency to the mean [^18^F]FDG uptake (Additional file 1: Fig. S1).

### Ex vivo validation

**Fig. 2:**
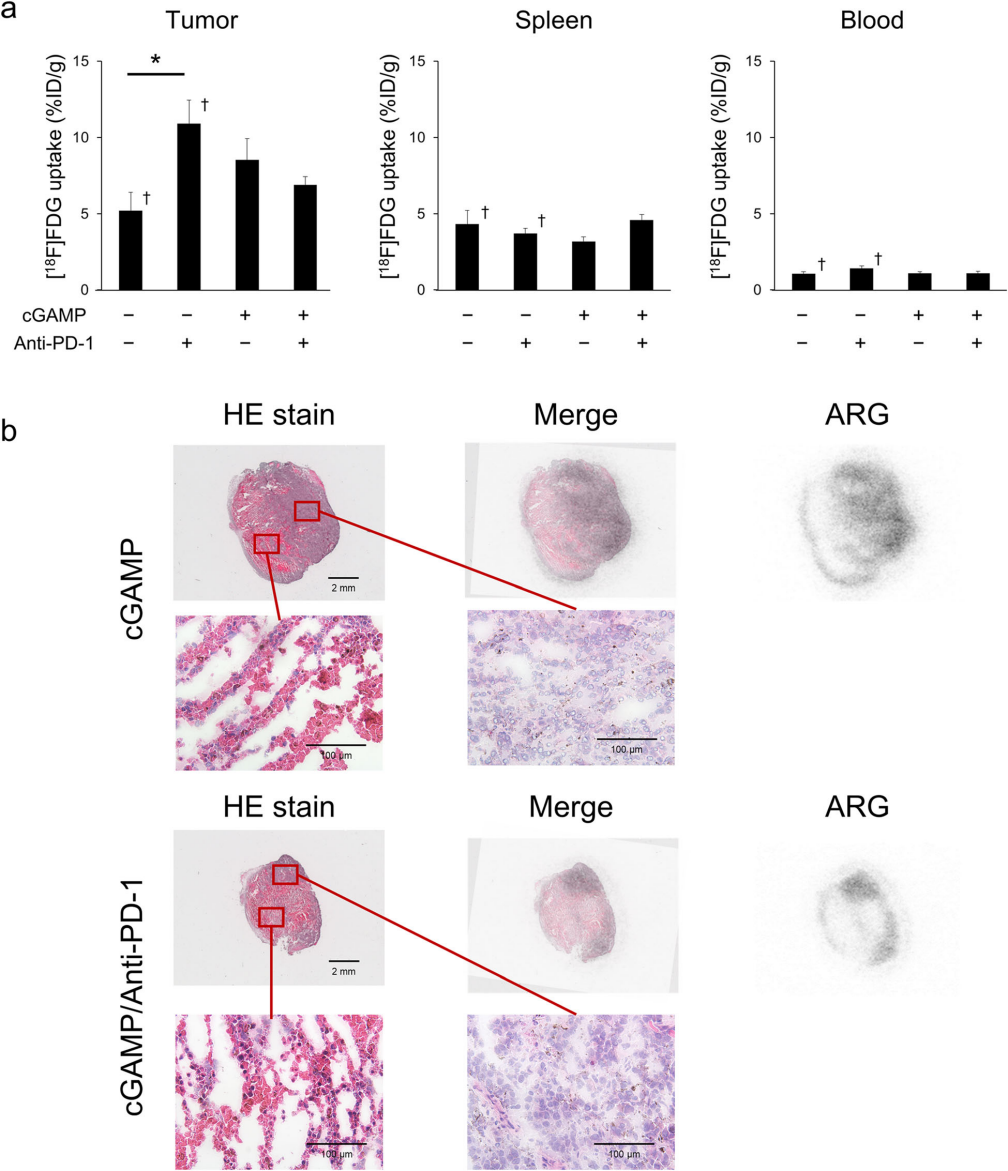
Ex vivo validation of [^18^F]FDG uptake. **a** Ex vivo biodistribution of tumors, spleens, and blood on day 7 (*n* = 6–7). Data of the non-treatment group and anti-PD-1-treated group were quoted from [11]. **b** HE-stained tissue sections and autoradiograms of the cGAMP-treated group and the cGAMP/anti-PD-1 combination group tumors. Data represent the mean ± SEM; *p < 0.05; † Adapted from [11]

Ex vivo validation measured using a gamma counter showed that [^18^F]FDG uptake in tumors was decreased in the order anti-PD-1-treated group, cGAMP-treated group, and cGAMP/anti-PD-1 combination group on day 7, although there were no significant differences between groups (Fig. 2a). However, cGAMP or PD-1 treatment did not change [^18^F]FDG uptake in the spleen and blood (Fig. 2a).

After PET-CT imaging, the intratumoral distribution of [^18^F]FDG was analyzed by autoradiography, and the autoradiographs were compared with HE staining. Figure 2b shows [^18^F]FDG uptake in non-necrotic areas and that anti-PD-1 or cGAMP treatment did not affect the histopathology.

### Effect of anti-PD-1 or cGAMP injection on immune cell populations in spleens and tumors

**Fig. 3:**
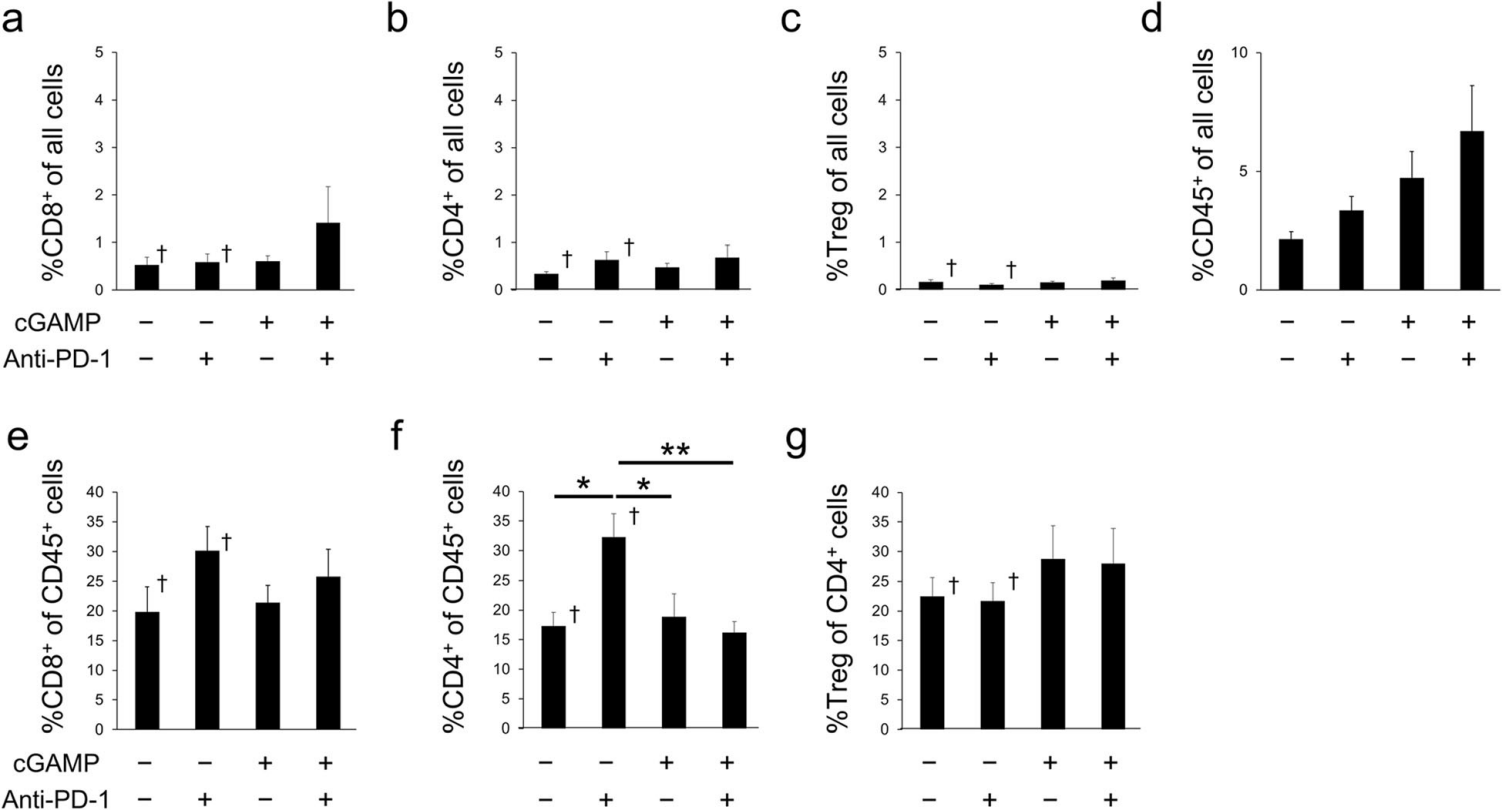
Flow-cytometry analysis of immune cell populations in tumors from the cGAMP-injected B16F10 model. Percentage of CD8^+^ cells (**a**), CD4^+^ cells (**b**), Foxp3^+^ cells (Treg, **c**), and CD45^+^ cells (**d**) in all cells in tumors on day 7 (*n* = 5–7). Percentage of CD8^+^ cells (**e**) and CD4^+^ cells (**f**) in CD45^+^ cells in tumors on day 7 (*n* = 5–7). Percentage of Foxp3^+^ cells (Treg, **g**) of CD4^+^ cells in tumors on day 7 (*n* = 5–7). Data of the non-treatment group and anti-PD-1-treated group were quoted from [11]. Data represent the mean ± SEM; *p < 0.05; **p < 0.01; † Adapted from [11]

To determine whether anti-PD-1 treatment affected immune cell populations in the cGAMP-injected model, flow-cytometry analysis was performed. cGAMP/anti-PD-1 combination treatment enriched the %CD8^+^ in all cells, but cGAMP injection did not affect regulatory T cell (Treg) or CD4^+^ T cell infiltration among all cells in tumors (Fig. 3a–c). The infiltration levels of CD45^+^ cells into tumors was increased in the order anti-PD-1-treated group, cGAMP-treated group, and cGAMP/anti-PD-1 combination group (Fig. 3d). Among CD45^+^ cells, anti-PD-1 and/or cGAMP treatment did not increase the frequency of CD8^+^ T cells, but the %CD4^+^ of CD45^+^ cells in anti-PD-1 treatment group tumors was significantly higher than in the other three groups (Fig. 3e, f). Furthermore, cGAMP treatment did not affect the infiltration level of Treg among CD4^+^ cells (Fig. 3g). The change in immune cell levels in the tumor was less than 10% (Fig. 3a–d). Also, immunohistochemistry shows CD3^+^ T cells infiltration into tumor in each group (Additional file 1: Fig. S2).

**Fig. 4:**
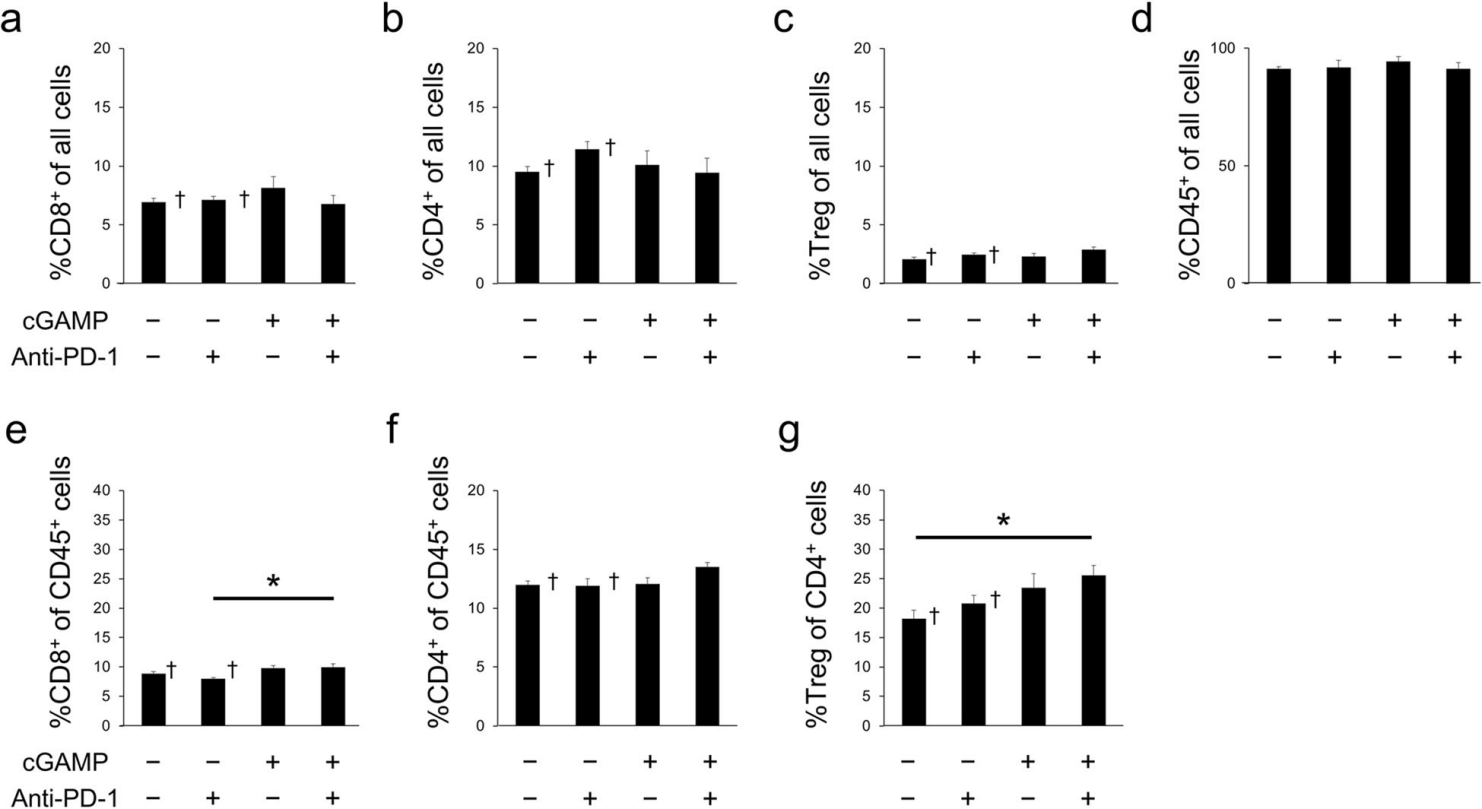
Flow-cytometry analysis of immune cell populations in spleens of the cGAMP-injected B16F10 model. Percentage of CD8^+^ cells (**a**), CD4^+^ cells (**b**), Foxp3^+^ cells (Treg, **c**), and CD45^+^ cells (**d**) in all cells in spleens on day 7 (*n* = 5–7). Percentage of CD8^+^ cells (**e**) and CD4^+^ cells (**f**) of CD45^+^ cells in spleens on day 7 (*n* = 5–7). Percentage of Foxp3^+^ cells (Treg, **g**) of CD4^+^ cells in spleens on day 7 (*n* = 5–7). Data of the non-treatment group and anti-PD-1 treated group were quoted from [11]. Data represent the mean ± SEM; *p < 0.05; † Adapted from [11]

However, anti-PD-1 or cGAMP treatment did not affect the percentage of CD8^+^ T cells, CD4^+^ T cells, Tregs, or CD45^+^ cells among all cells in the spleen (Fig. 4a–d). In addition, the %CD8^+^ of CD45 cells in the spleens of the cGAMP/anti-PD-1 combination treated group was significantly higher than that of the anti-PD-1 alone treated group, but the %CD4^+^ of CD45^+^ cells in the spleen was similar in all four groups (Fig. 4e, f). Also, the %Treg of CD45^+^ cells in the spleens of the cGAMP/anti-PD-1 combination treated group was significantly higher than that of the anti-PD-1 alone treated group (Fig. 4g). However, the change in immune cell levels in the spleen was less than 5% (Fig. 4a–d).

### Effect of anti-PD-1 or cGAMP injection on glucose metabolism in spleens and tumors

**Fig. 5:**
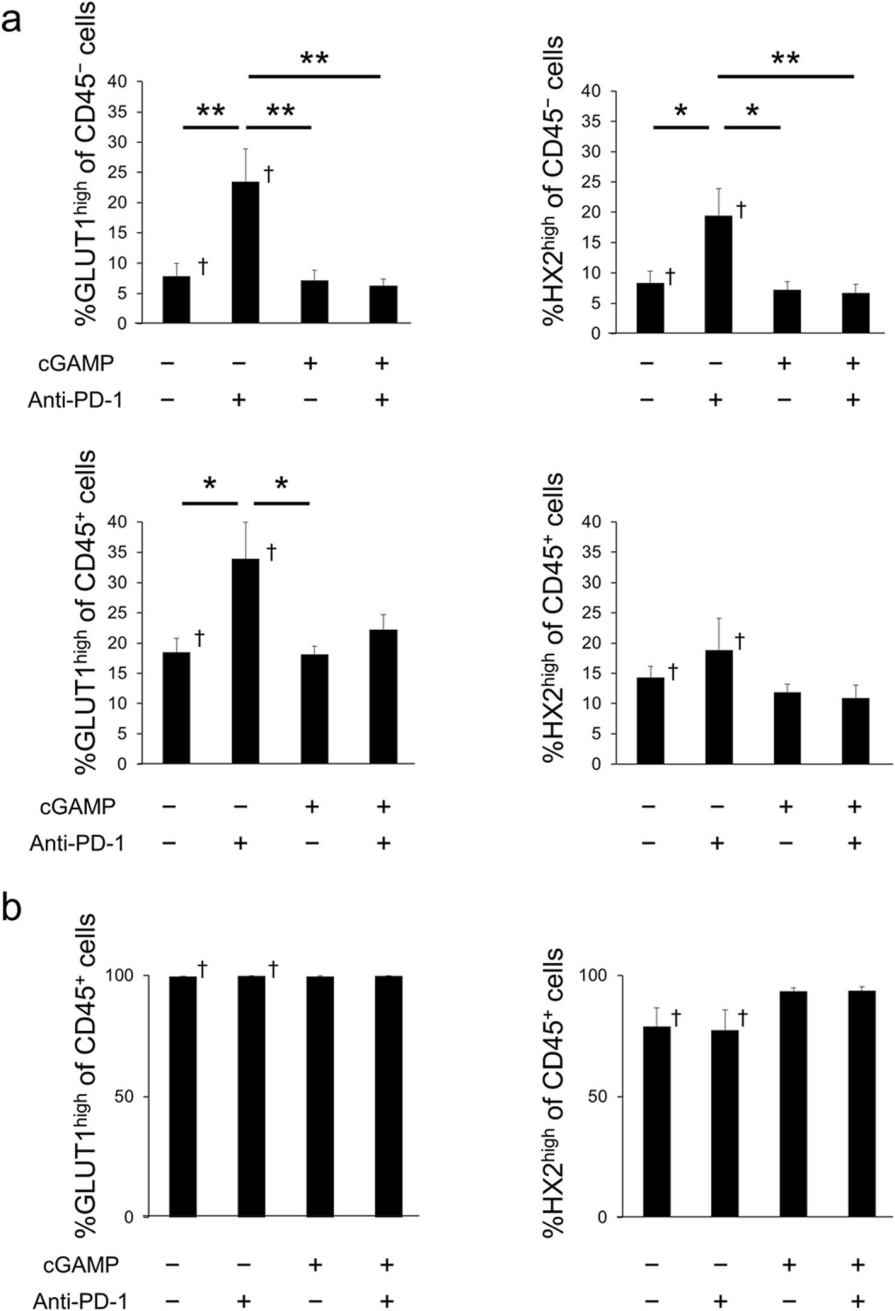
Flow-cytometry analysis of glycolysis in the cGAMP-injected B16F10 model. **a** Percentage of GLUT1 (left) and HX2 (right) high expressing cells in CD45^−^ cancer cells (top) or CD45^+^ immune cells (bottom) in tumors on day 7 (*n* = 5–7). Data of the non-treatment group and anti-PD-1-treated group were quoted from [11]. **b** Percentage of GLUT1 (left) and HX2 (right) high expressing cells in CD45^+^ immune cells in spleens on day 7 (*n* = 5–7). Data of the non-treatment group and anti-PD-1-treated group were quoted from [11]. Data represent the mean ± SEM; *p < 0.05; **p < 0.01; † Adapted from [11]

The expressions of glycolysis markers (GLUT1 and HX2) were measured by flow-cytometry analysis. The cGAMP/anti-PD-1 combination group or cGAMP alone treated group had significantly lower levels of GLUT1^high^ cells/HX2^high^ cells of CD45^−^ cancer cells in tumors compared with the anti-PD-1 treated group (Fig. 5a). Furthermore, the cGAMP/anti-PD-1 combination group or cGAMP alone treated group had lower levels of GLUT1^high^ cells/HX2^high^ cells of CD45^+^ immune cells in tumors compared with the anti-PD-1 treated group (Fig. 5a). In spleens, the %GLUT1^high^ or HX2^high^ of CD45^+^ cells was similar in all four groups (Fig. 5b) and blood sugar levels in all groups were normal (Additional file 1: Table S1).

## Discussion

In this study, cGAMP alone or cGAMP/anti-PD-1 combination treated tumors had a smaller volume compared with the non-treated or anti-PD-1 alone treated tumor on day 7 (Fig. 1b). Among these groups, cGAMP/anti-PD-1 combination therapy was the most effective. The infiltration of CD45^+^ cells and CD8^+^ cells were elevated in the cGAMP/anti-PD-1 combination group (Fig. 3a, d). Furthermore, cGAMP treatment enriched CD45^+^ T cells. Thus, cGAMP injection promoted the recruitment of immune cells at the tumor site in this study as previously reported [15], and induced anti-tumor responses.

Previous experiments by our group showed that [^18^F]FDG uptake in anti-PD-1 treatment group tumors was higher than that in non-treatment group tumors following treatment, as shown by PET-CT and ex vivo validation, on day 7 [11]. In this study, we compared the anti-PD-1 alone treated group with the cGAMP/anti-PD-1 combination treated group, for the purpose of revealing the effect of anti-PD-1 treatment on tumor glycolysis in a highly immune-activated tumor. The present study demonstrated that [^18^F]FDG uptake in tumors in the cGAMP/anti-PD-1 combination group was lower than that in the anti-PD-1 treatment group tumors on day 7, as shown by PET-CT (Fig. 1d). Furthermore, the mean [^18^F]FDG uptake within the MTV or TLG, excluding necrotic areas, had a similar tendency. Moreover, as shown by ex vivo validation (Fig. 2a), [^18^F]FDG uptake in tumors was decreased in the order anti-PD-1 treated group, cGAMP treated group, and cGAMP/anti-PD-1 combination group on day 7. This order corresponded to the order of CD45^+^ cell infiltration level and tumor reduction effects. That is, the stronger the anti-tumor effect, the greater the decrease in [^18^F]FDG uptake in tumors. Thus, our results suggest that if immune responses in tumors are higher than a certain level, glucose uptake in tumors is reduced depending on that level. Such a change in glucose uptake might be caused by the difference in infiltration or activation level of immune cells between the anti-PD-1 treated group and the cGAMP/anti-PD-1 combination group.

In contrast to chemotherapies that directly target cancer cells, anti-PD-1 therapy influences the immune system. Thus, anti-PD-1 treatment should affect metabolism in the TME including cancer cells and immune cells among others. Recent investigations reported that glucose consumption by tumors metabolically restricted T cells, blocking PD-L1 directly on tumors to dampen glycolysis by inhibiting mTOR activity, and decreasing the expression of glycolysis enzymes [9]. Another study reported that the upregulation of glycolysis was a critical step in the activation of adaptive immune cells [21]. Therefore, it is necessary to determine the effect of anti-PD-1 treatment on the uptake of [^18^F]FDG, a marker of glucose uptake in cells (e.g., effector immune cells and cancer cells). Clinical studies reported that immune checkpoint inhibitors increased [^18^F]FDG uptake; however, other studies showed the opposite effect [22–25]. In this study, Fig. 3 shows anti-PD-1 and/or cGAMP treatment changed the infiltration level of immune cells into tumors as well as the immune cell composition in tumors. Especially, the %CD4^+^ T cells in CD45^+^ cells was significantly changed in response to anti-PD-1 and/or cGAMP treatment. Because CD4^+^ T cells produce cytokines that induce glycolysis in cancer cells [26], this cellular composition may be an important factor affecting glycolysis of cancer cells. In this study, [^18^F]FDG uptake in tumors in the cGAMP/anti-PD-1 combination group was lower than that in the anti-PD-1 treatment group tumors on day 7, although the difference was not significant (ex vivo validation: *p* = 0.101) (Fig. 2a). Thus, [^18^F]FDG uptake in tumors might be affected by the tumor immune microenvironment including immune cell infiltration, composition, and activation status. This might explain why anti-PD-1 therapy increased [^18^F]FDG uptake in this study, but decreases it in clinical studies.

We performed flow-cytometry analysis in the cGAMP-injected B16F10 model focusing on GLUT1 and HX2 to clarify the mechanism of the change in [^18^F]FDG uptake in tumors (Fig. 5a). Among CD45^−^ cancer cells, GLUT1^high^ cells and HX2^high^ cells in tumors were significantly lower in the cGAMP/anti-PD-1 combination group compared with the anti-PD-1 treated group on day 7. These results of GLUT1 and HX2 expression level that were analyzed by flow-cytometry mostly corresponded with the ex vivo validation results of [^18^F]FDG uptake, but were not completely concordant with them. This should be because flow-cytometry analysis could exclude the necrotic regions, while the necrotic regions affect the results on [^18^F]FDG uptake in tumors (Figs. 1d and 2d). But we observed that [^18^F]FDG uptake in tumors in the cGAMP/anti-PD-1 combination group was lower than that in the anti-PD-1 treatment group tumors and these results corresponded with GLUT1 (and HX2) expression level in cancer cells. Therefore, immune responses induced by anti-PD-1 or cGAMP treatment affected glucose metabolism in cancer cells, thereby changing the [^18^F]FDG uptake in tumors. These results complement those reported by Chang et al. who found that blocking PD-L1 on tumors dampened glycolysis by inhibiting mTOR activity and decreasing the expression of glycolysis enzymes [9]. Previous experiments by our group showed that changes in [^18^F]FDG uptake occurred only in tumors and not in the spleen [11]. In addition, cGAMP or PD-1 treatment of the cGAMP-injected B16F10 model did not change [^18^F]FDG uptake in the spleen (Fig. 2a). Moreover, flow-cytometry analysis revealed that anti-PD-1 or cGAMP treatment resulted in a minimal change in the composition of immune cells and a negligible effect on glucose metabolism by CD45^+^ cells in the spleen (Fig. 5). These results supplement the finding that increased [^18^F]FDG uptake in tumors was not caused by immune cells but rather by cancer cells.

PD-1 blockade represents an innovative mechanism of action, and is more effective at improving survival time with less toxicity compared with conventional chemotherapies for some types of cancer. However, PD-1 inhibitors are not effective in all patients because the therapeutic effects depend on individual immunity and mutation status of cancer genes [5, 6]. Therefore, it is necessary to monitor or predict responses to anti-PD-1 therapies. [^18^F]FDG is a widely used radiotracer for cancer imaging and it is important to evaluate the value of [^18^F]FDG-PET for the monitoring or prediction of the effects of anti-PD-1 therapies. However, the effect of anti-PD-1 therapy on [^18^F]FDG uptake is not clear. The current study suggests that the tumor immune microenvironment during anti-PD-1 treatment affects glucose metabolism in cancer cells. This suggests that anti-PD-1 treatment has various effects on [^18^F]FDG uptake related to the differences in individual patient immunity.

It is desirable to combine metabolic imaging to monitor anti-PD-1 therapy with the infiltration levels of immune cells in tumors. A recent study reported that anti-^89^Zr-radiolabeled CD4 and CD8 immuno-PET reagents represented a powerful resource to monitor T cell expansion, localization, and novel engraftment protocols [27]. Another study showed that ^89^Zr-labeled anti-CD8 immuno-PET was a sensitive tool for detecting changes in systemic and tumor-infiltrating CD8 expression in preclinical syngeneic tumor immunotherapy models including immune checkpoint blockade antibody therapy (anti-PD-L1) [28]. By using a combination of metabolic imaging and immuno-imaging techniques that are being rapidly developed, we might be able to evaluate both metabolic status and immune activation, allowing the monitoring of anti-PD-1 treatment effects. Further studies are needed to evaluate the value of [^18^F]FDG-PET for monitoring or prediction of responses to anti-PD-1 therapies; for example, by using highly immunogenic mouse models, considering the tumor central area with less immune cell infiltration and the invasive margin with high immune cell infiltration separately, and conducting long-term experiments and combining [^18^F]FDG-PET and immune-PET imaging.

## Conclusion

[^18^F]FDG uptake in cancer cells after anti-PD-1 therapy might be affected by the tumor immune microenvironment including immune cell infiltration, composition and activation status.

## Supplementary information

**Additional file 1: Figure S1.** Analysis of [^18^F]FDG-PET data to exclude necrotic regions of the tumor. **a** Mean_30%_ [^18^F]FDG uptake calculated by PET-CT images in tumors on day 7 (*n* = 6–7). Data of the non-treatment group and anti PD-1 treated group were quoted and reanalyzed from [11]. **b** TLG calculated by PET-CT images in tumors on day 7 (*n* = 6–7). Data of the non-treatment group and anti PD-1 treated group were adopted and reanalyzed from [11]. Data represent the mean ± SEM. **Figure S2.** CD3 staining of tumor samples in each group. **Table S1.** Blood glucose levels on day 7 (*n* = 4). Data represent the mean ± SEM.

## Data Availability

Data sharing not applicable to this article as no datasets were generated or analyzed during the current study.

## Abbreviations

%ID/g: Percentage-injected dose per gram of tissue
[^18^F]FDG: 2-Deoxy-2-[^18^F]fluoro-*D*-glucose
cGAMP: Cyclic dinucleotide GMP-AMP
CT: Computed tomography
GLUT1: Glucose transporter 1
HX2: Hexokinase II
MTV: Metabolic tumor volume
PD-1: Programmed cell death 1
PET: Positron emission tomography
STING: Stimulator of IFN genes
TLG: Total legion glycolysis
TME: Tumor microenvironment
Treg: Regulatory T cell
VOI: Volume of interest
