# Supplementary material for: Influence on [18F]FDG uptake by cancer cells after anti-PD-1 therapy in an enforced-immune activated mouse tumor
Source: EJNMMI Res. 2020 Mar 19;10:24. doi: 10.1186/s13550-020-0608-4 (PMC7080890; doi:10.1186/s13550-020-0608-4)
Supplement: Supplementary file 1 — Additional file 1: Figure S1. Analysis of [18F]FDG-PET data to exclude necrotic regions of the tumor. a Mean30% [18F]FDG uptake calculated by PET-CT images in tumors on day 7 (n = 6–7). Data of the non-treatment group and anti PD-1 treated group were quoted and reanalyzed from [11]. b TLG calculated by PET-CT images in tumors on day 7 (n = 6–7). Data of the non-treatment group and anti PD-1 treated group were adopted and reanalyzed from [11]. Data represent the mean ± SEM. Figure S2. CD3 staining of tumor samples in each group. Table S1. Blood glucose levels on day 7 (n = 4). Data represent the mean ± SEM. [file 13550_2020_608_MOESM1_ESM.pdf]

Supporting information

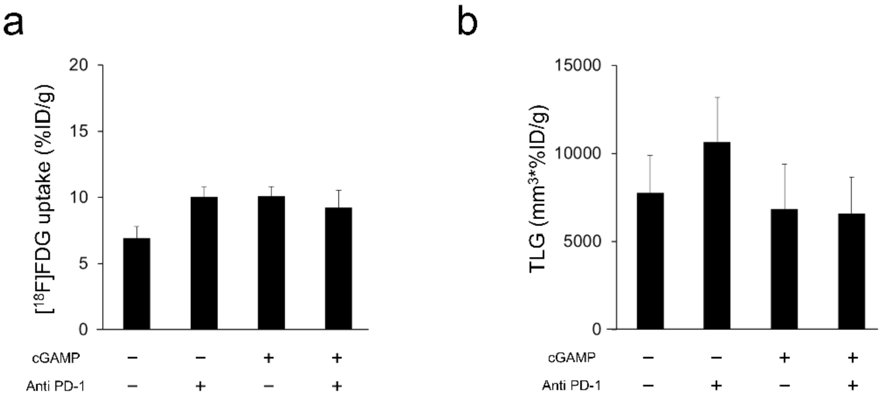

Fig. S1

Analysis of [^{18}F]FDG-PET data to exclude necrotic regions of the tumor. **a** Mean$_{30\%}$ [^{18}F]FDG uptake calculated by PET-CT images in tumors on day 7 (n = 6-7). Data of the non-treatment group and anti PD-1 treated group were quoted and reanalyzed from [11]. **b** TLG calculated by PET-CT images in tumors on day 7 (n = 6-7). Data of the non-treatment group and anti PD-1 treated group were adopted and reanalyzed from [11]. Data represent the mean ± SEM.

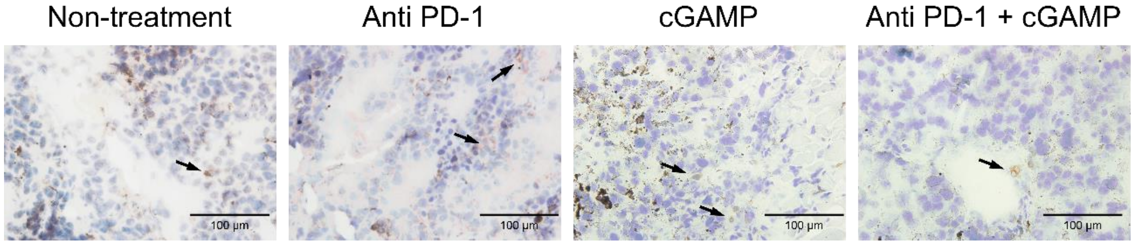

Fig. S2

CD3 staining of tumor samples in each group.

Table S1

Non-treatment	Anti PD-1	cGAMP	Anti PD-1 + cGAMP
86.8 ± 3.7 mg/dL	101.3 ± 8.2 mg/dL	74.0 ± 12.3 mg/dL	76.0 ± 21.9 mg/dL

Blood glucose levels on day 7 (n = 4). Data represent the mean ± SEM.
